# Efficacy of intravenous eptifibatide in primary percutaneous coronary intervention patients

**DOI:** 10.25122/jml-2021-0035

**Published:** 2021

**Authors:** Rozita Jalalian, Samad Golshani, Hossein Farsavian, Mahsa Vatani, Ali Asghar Farsavian

**Affiliations:** 1. Department of Cardiology, Faculty of Medicine, Cardiovascular Research Center, Mazandaran University of Medical Sciences, Sari, Iran; 2. Department of Vascular Surgery, Mazandaran University of Medical Sciences, Sari, Iran; 3. Department of Cardiology, Mazandaran University of Medical Sciences, Sari, Iran

**Keywords:** eptifibatide, heparin, primary PCI

## Abstract

Early and complete restoration of blood flow in closed coronary arteries is the main goal in treating patients with myocardial infarction. Primary angioplasty is not always successful in establishing myocardial blood flow. Although the strategy of adding eptifibatide leads to better blood flow, its value as part of a routine strategy is questionable. Therefore, this study was performed to evaluate the efficacy of intravenous eptifibatide in primary percutaneous coronary intervention (PCI) patients. This clinical, randomized, double-blind trial was performed on patients aged 20-80 years undergoing primary PCI. The patients were selected for study by convenience sampling and were randomly divided into two equal groups. The first group was treated with intravenous eptifibatide immediately before angioplasty with heparin. The second group received only coronary angioplasty with heparin. After data collection, statistical analysis was performed using the Statistical Package for the Social Sciences (SPSS) software, version 16. A total of 104 patients were enrolled in the study, and there were no statistically significant differences in terms of age (P=0.188), gender (P=0.345), risk factor (P>0.05), or history of PCI (P=0.199). Mean thrombolysis in myocardial infarction (TIMI) score was not significant between the two groups after receiving the drug and performing angioplasty (P>0.05), and the rate of ejection fraction was 46.33±6.69 in patients receiving eptifibatide and 47.54±4.67 in the heparin group, which was not statistically significant (P=0.884). We found that eptifibatide improves clinical indexes in patients undergoing primary PCI, but these differences were not significant in the two groups.

## INTRODUCTION

Coronary artery disease is one of the most common causes of death worldwide. Nearly one-third of Americans over the age of 35 die from this disease. Coronary artery disease refers to the pathological processes of atherosclerosis that affect the coronary arteries [1, 2]. Treatment of these patients can be done through medication or invasive strategy, and decisions are made for each person according to personal circumstances [3, 4]. Initially, complete and stable coronary blood flow is an essential criterion for standard treatment of patients suffering from myocardial infarction with ST-segment elevation. Fibrinolytic treatment will reduce mortality in patients immediately after the onset of symptoms [5], but there are some limitations. Fibrinolytic treatment normalizes coronary blood flow in only 60% of patients and has a high risk of bleeding and hemorrhagic events. The treatment of choice for patients with ST-Elevation Myocardial Infarction (STEMI) is primary percutaneous coronary intervention (PCI), in which the rate of bleeding is lower [6]. However, the movement of atherothrombotic material during primary PCI is very common in STEMI patients. This can lead to obstruction of the distal arteries and improper myocardial perfusion, which is associated with larger infarcts, impaired ventricular function, and increased mortality.

Thrombosis and vascular debris may move and form plaques in the microvascular system, causing myocardial dysfunction and necrosis [7]. There are many therapeutic strategies to improve microvascular perfusion after primary PCI, such as thrombus aspiration, GP2b/3a administration (intracoronary bolus), and others. Eptifibatide is an antiplatelet drug that reversibly inhibits the binding of fibrinogen, Von Willebrand factor, and other adhesive molecules to the platelet glycoprotein receptor IIb/IIIa; it is usually used in combination with aspirin and a p2y12 inhibitor and as an anti-coagulant treatment for acute coronary syndrome, myocardial infarction, and patients undergoing PCI. The half-life of the drug is about 2.5 hours, and about 25% of it can bind to plasma proteins. The drug metabolite is about 5% of the total body clearance, and it is excreted through urine [9]. Similar studies have investigated the long-term efficacy of eptifibatide on the ejection fraction (EF) and myocardial perfusion [5, 6, 10]. Therefore, this study was performed to evaluate the effectiveness of intravenous eptifibatide in improving EF and myocardial perfusion in primary PCI patients and to assess the rate of bleeding complications of this drug.

## MATERIAL AND METHODS

This clinical, randomized, and double-blind trial was performed on patients aged 20–80 years referred to Mazandaran Heart Hospital, Iran, who underwent primary PCI with an acute STEMI clinical picture from July 2018 to April 2019. Patients were selected by convenience sampling and were randomly divided into one of two intervention groups by cardiovascular assistants. These patients did not know which group they were in; then, patients who met the inclusion criteria were included in the study.

### Inclusion criteria

- Typical ischemic chest pain over 30 minutes;
- ST-segment elevation greater than one millimeter in at least two leads;
- All STEMI patients who were candidates for primary PCI;
- Myocardial infarction that did not last more than 12 hours.

### Exclusion criteria

- Rescue PCI after treatment with thrombolytics;
- Need for emergency Coronary artery bypass grafting (CABG);
- Cardiogenic shock;
- Life expectancy less than 6 months;
- Patient dissatisfaction;
- Age under 18 years and over 80 years;
- Evidence of previous myocardial infarction seen on an electrocardiogram;
- Contraindications to the use of eptifibatide;
- Left Bundle Branch Block (LBBB);
- Observing TIMI grade 3 flow during primary angiography;
- Kidney failure;
- Use of GP2b/3a drugs over the last two weeks.

### Sample size

The sample size of this research was calculated based on the following statistical formula:


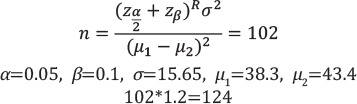


By considering a 20% of sample loss, mean EF before and after treatment, and reference results [10], the sample size was calculated to be 124 individuals.

### Procedure

All patients received 300 mg of chewable aspirin and 600 mg of clopidogrel before the procedure. In all patients, a percutaneous femoral artery approach was used for catheterization. In both intervention groups, wiring of the caliper vessel was also performed, followed by balloon dottering. Predilation and thrombosuction were performed if necessary.

Group 1 included patients with TIMI 0-1; they received 100 units of heparin/kg and then underwent balloon dottering after wiring of the culprit vessel; if necessary, low-pressure balloon inflation was performed, then stenting was done. Patients in group 2 received 50 to 70 μg/kg of heparin and 180 μg/kg of eptifibatide by intravenous bolus were injected. Wiring of the culprit vessel and, if necessary, low-pressure balloon inflation were performed. Then, stenting was performed, and eptifibatide was continued as a 12-hour infusion. From all cardiac referrals, a 12-lead electrocardiogram (ECG) was taken at baseline and 90 minutes after PCI in the coronary care unit (CCU). Both electrocardiograms were examined by a blind observer. In all patients, echocardiography was performed by one person at the beginning of admission and 3–4 days after PCI. 2D and Doppler echo parameters were measured using the standard methods. All volumes were normalized based on the body surface index. Regional wall motion was performed using a 17-segment model approved by the American Society of Echocardiography and the European Association of Echocardiography. For 2D-strain analysis, a 17-segment model of the left ventricle (LV) was prepared from apical 2, 3, and 4 chambers views and parasternal short-axis (PSAX) view at the basal, middle, and apical levels by automatic software. Positive or negative strain value was defined during systole. The global longitudinal strain (GLS), global circumferential strain (GCS), global radial strain (GRS) values were obtained by calculating the mean peak systolic strain value in the 18-segment model. In the studied patients, Ck-Mb and troponin were measured time at admission and 24 hours later.

### Statistical analysis

Data were described based on the mean and standard deviation for quantitative variables, while numbers and percentages were considered for qualitative variables. The relative frequency of complications between the two groups was assessed by the Chi-Square test. Statistical analysis was performed after entering the data into the Statistical Package for the Social Sciences (SPSS) software, version 16. A significance level of 0.05 was considered.

## RESULTS

A total of 104 patients were included in the study, of whom 59 were in the heparin group and 45 in the eptifibatide group. The mean age was 56.53±8.1 in the heparin group and 55.53±10.1 in the eptifibatide group. There was no statistically significant relationship between the two groups in terms of age (P=0.188). The eptifibatide group consisted of 34 (75.6%) males and 11 (24.4%) females. The heparin group included 49 (83.1%) males and 10 (16.9%) females. There was no statistically significant difference between the two groups in terms of gender (P=0.345).

**Figure 1:**
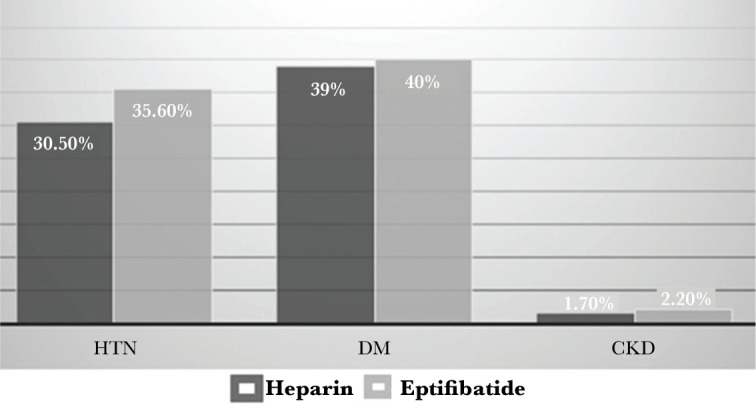
Disease history of study participants.

In the eptifibatide group, history of diabetes, hypertension, and chronic kidney disease were revealed for 18 patients (40%), 16 patients (35.6%) and 1 patient (2.2%) respectively, while 27 (60%), 29 (64.4) and 44 patients (97.8%) had no history of these diseases, respectively. In the heparin group, history of diabetes, hypertension, and chronic kidney disease were recorded for 23 patients (39.4%), 18 (30.5%) and 1 patient (1.7%), respectively, whereas 36 patients (60.6%), 41 patients (69.5 %) and 58 patients (98.3%) had no history of these diseases, respectively which did not show a statistically significant difference (P=0.916, P=0.587, P=0.846, respectively) (Figure 1).

**Figure 2:**
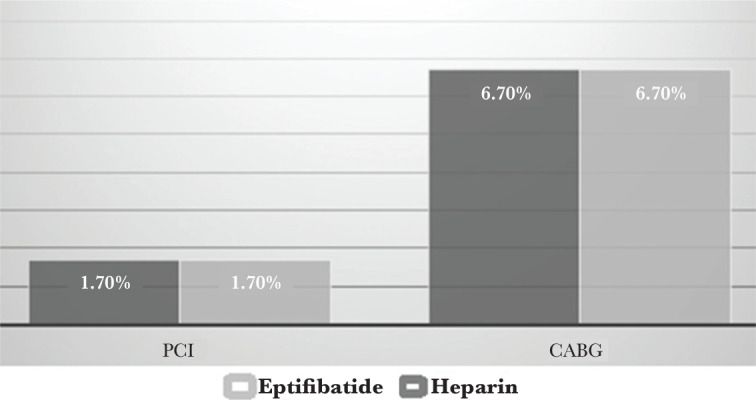
Evaluation of participants in terms of PCI.

Regarding the history of PCI in the eptifibatide group, 3 patients (6.7%) had a history of PCI, and 42 patients (93.3%) had no history. In the heparin group, 1 patient (1.7%) had a history of PCI and 58 patients (98.3%) had no PCI history. Overall, there was no statistically significant difference between the two groups (P=0.191) (Figure 2).

Participants were evaluated for chest pain before and after PCI. In the eptifibatide and heparin groups, 28 patients (62.3%) and 53 patients (89.9%) had chest pain before PCI, respectively, and 17 patients (37.7%) and 6 patients (10.1%) had no chest pain before PCI. After PCI, 45 patients (100%) and 58 patients (98.1%) did not have chest pain, and only one person in the heparin group had chest pain after PCI; these findings did not show a statistically significant difference in terms of chest pain before and after PCI (P=0.510, P=0.734). Participants were evaluated for wall motion index before and after the intervention in two treatment groups. The study results showed that the mean wall motion index before the intervention was 1.18±0.19 in the heparin group and 1.22±0.21 in the eptifibatide group. After the intervention, the wall motion index was 1.5±0.23 in the heparin group and 1.22±0.21 in the eptifibatide group. There was no statistically significant difference between the results before and after the wall motion index (P=0.061, P=0.08).

**Figure 3:**
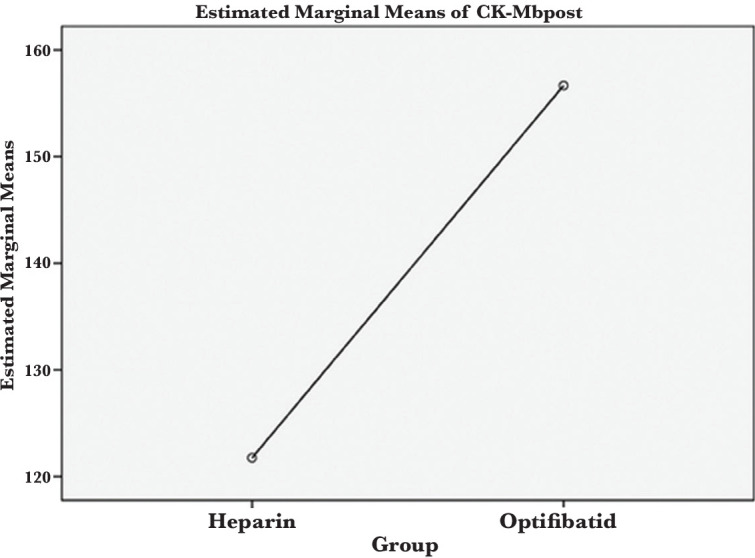
Evaluation of participants regarding CK-MB values after intervention in the treatment groups.

Our findings showed that patients in the eptifibatide group had lower CK-MB values (average of 156.49±143.447) than the heparin group who had an average of 121.88±103.155, but the two groups did not show a statistically significant difference in CK-MB levels before and after the intervention (Table 1 and Figure 3; P>0.05).

**Table 1:** Evaluation of participants regarding CK-MB values.

Variable	Before intervention		After intervention	
	Heparin	Eptifibatide	Heparin	Eptifibatide
**CK-MB**	121.88±103.155	26.22±10.282	29.15±13.513	156.49±143.447
	P=0.086		P=0.948	

**Figure 4:**
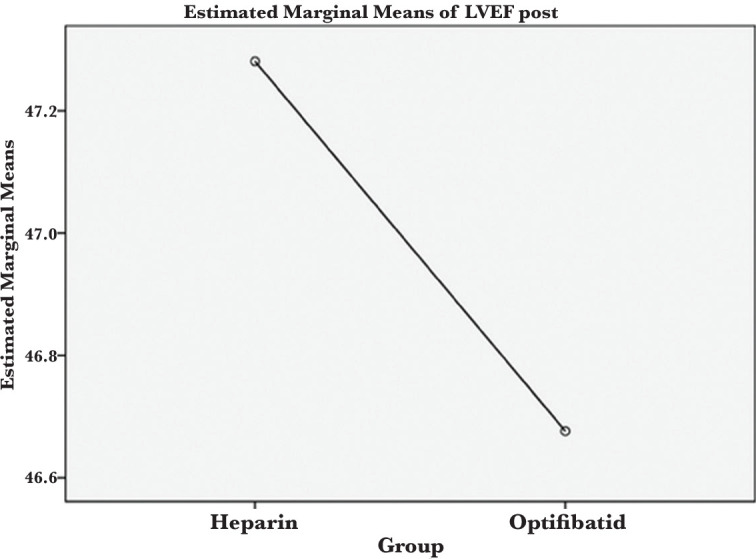
Rate of ventricular ejection fraction after the intervention.

Left ventricular ejection fraction (LVEF) before intervention in patients in the heparin group was 40.59±5.5. In the eptifibatide group, it was 39.56±8.5%. After the intervention, LVEF was 47.54±4.6 in the heparin group and 46.33±6.6 in the eptifibatide group. No significant difference was found between the two groups regarding LVEF before and after the intervention (P=0.948, P=0.884; Figure 4).

The results showed that sum ST resolution above 70% was seen in 74.5% cases in the heparin group and 73.3% cases in the eptifibatide group. Furthermore, a sum ST Resolution of 50% was found to be 88.1% and 91.1% in the heparin and eptifibatide groups, respectively, which was not statistically significant between the two groups (P=0.341; Table 2).

**Table 2:** Evaluation of participants in terms of sum ST resolution.

		Group		P-Value
Sum ST resolution		Eptifibatide	Heparin	
**>70%**	**Frequency**	33	44	0.348
	**Percent**	73.3%	74.5%	
**50%**	**Frequency**	41	52	0.341
	**Percent**	91.1%	88.1%	

Participants were examined for mitral regurgitation before and after the intervention in two groups (Table 3). The results revealed no statistically significant difference between the two groups in terms of MR before and after the intervention (P>0.05).

**Table 3:** Evaluation of participants for mitral regurgitation before and after intervention in the two groups.

MR	Before intervention		After intervention	
	Heparin	Eptifibatide	Heparin	Eptifibatide
**Mild**	43 (72.9%)	31 (61.9%)	29 (49.2%)	18 (40%)
**Moderate**	2 (3.4%)	4 (8.91%)	1 (1.76%)	0 (0%)
**Severe**	1 (1.7%)	2 (4.4%)	0 (0%)	1 (1%)
	P=0.514		P=0.406	

The results of Table 4 showed that there was no significant difference between the two groups in terms of TIMI flow before and after the intervention based on the territory.

**Table 4:** Evaluation of participants in terms of TIMI flow based on the territory.

TIMI post *Group* territory cross-tabulation						
Territory				Group		Total
				Heparin	Eptifibatide	
**Group 1**						
**(Patients with STEMI in the LAD territory)**	**TIMI post**	**2**	Count	6	7	13
			% within group	20.7%	26.9%	23.6%
		**3**	Count	23	19	42
			% within group	79.3%	73.1%	76.4%
	**Total**		Count	29	26	55
			% within group	100.0%	100.0%	100.0%
**Group 2**						
**(Patients with STEMI in a**						
**non-LAD territory)**	**TIMI post**	**2**	Count	9	5	14
			% within group	30.0%	26.3%	28.6%
		**3**	Count	21	14	35
			% within group	70.0%	73.7%	71.4%
	**Total**		Count	30	19	49
			% within group	100.0%	100.0%	100.0%
**Total**	**TIMI post**	**2**	Count	15	12	27
			% within group	25.4%	26.7%	26.0%
		**3**	Count	44	33	77
			% within group	74.6%	73.3%	74.0%
	**Total**		Count	59	45	104
			% within group	100.0%	100.0%	100.0%

LAD – left anterior descending artery; STEMI – ST-elevation myocardial infarction; TIMI post – TIMI flow grade after primary percutaneous coronary intervention.

## DISCUSSION

Early and complete restoration of blood flow in closed coronary arteries is the main goal in treating patients with myocardial infarction. Primary angioplasty is not always successful in restoring myocardial blood flow, despite success in the opening of the epicardial coronary arteries. Eptifibatide is a glycoprotein IIb/IIIa receptor antagonist. Although the strategy of adding eptifibatide leads to better blood flow, its value as part of the routine strategy is questionable, according to the results of previous studies [11, 12]. Therefore, this study aimed to evaluate the effectiveness of intravenous eptifibatide in improving myocardial perfusion and the rate of hemorrhagic complications in primary PCI patients. The results of the present study demonstrated that eptifibatide has no significant advantage in the coronary artery flow compared to the usual treatment of patients. In the present study, no significant difference was found in terms of TIMI flow between the eptifibatide and heparin treatment groups. However, Gibson *et al*. reported that early administration of eptifibatide was associated with improved epicardial flow and increased myocardial perfusion in patients with acute myocardial infarction, which was not consistent with the results of the present study [5].

Contrary to the results of the present study, other studies have suggested that the administration of eptifibatide is associated with improved myocardial perfusion [5, 13]. In other words, this drug is effective in correcting perfusion parameters and the results of laboratory studies, which show an increase in clot dissolution when increasing eptifibatide concentration. In 2011, Mahmoudi *et al*. reported that the eptifibatide group showed a 6-month benefit in terms of mortality compared to the adjuvant group at Washington Hospital [14].

In the current study, the percentage of ST-segment resolution after treatment in the eptifibatide group was higher than in the heparin group. This means that the effect was higher in the eptifibatide group, but no statistically significant difference was found between the two groups in this regard. In fact, most of the variables in the intervention group were better, but their differences were not significant compared to the control group.

Zeymer *et al*. compared the effects of early and delayed injection of eptifibatide in patients with STEMI; out of a total of 102 patients, 2 in each group had bleeding complications, but no difference has been found between the two groups, which were consistent with the results of our study. Also, no statistical difference was found between the two groups in terms of ST-segment resolution rate, and TIMI flow rate was similar in both groups after angioplasty, results which are somewhat consistent with our findings [15].

The Joint Canada/United States Survey of Health (JCUSH) divided patients undergoing elective or emergency PCI into two groups, one receiving placebo and the other receiving eptifibatide along with aspirin and heparin. Eptifibatide infusion was continued for 24–28 hours after angioplasty or until the patient was discharged from the hospital. The results of the aforementioned study showed that the rate of major and minor bleeding was higher in the eptifibatide group, which was not consistent with the results of our study [16]. In the study of Le May *et al*., it was found that the rate and frequency of bleeding complications were significantly higher in the eptifibatide group than in the control group. The reasons for the low rate of bleeding in the present study include the accuracy and skill of the operator, the careful selection of patients based on stringent inclusion and exclusion criteria, and the infusion of eptifibatide with a shorter duration (12 hours) [17].

In another study by Soon *et al*., two groups of patients (who received intracoronary bolus and intravenous injection) were evaluated for myocardial infarction. Patients in the intervention group received an eptifibatide intracoronary bolus dose, and patients in the control group received an eptifibatide infusion for 18 hours after PCI. The results of the study revealed that both groups were in a favorable prognosis in terms of angiographic findings [18]. In addition, Gibson *et al*. compared the effect of intracoronary versus intravenous eptifibatide during PCI. The results of their study showed that epicardial TIMI flow had a significant increase in patients receiving intravenous eptifibatide compared to the intracoronary route [5], indicating that eptifibatide increases myocardial perfusion.

## CONCLUSION

Eptifibatide improves clinical indexes in patients undergoing primary PCI, but these rates were not found to be statistically significant. Thus, further studies are needed to confirm or refute these data.
